# Estimating the Slope Safety Factor Using Simple Kinematically Admissible Solutions

**DOI:** 10.3390/ma16227074

**Published:** 2023-11-07

**Authors:** Kamil Bacharz, Magdalena Bacharz, Wiesław Trąmpczyński

**Affiliations:** Department of Materials Strength and Building Structures, Kielce University of Technology, 25-314 Kielce, Poland; kbacharz@tu.kielce.pl

**Keywords:** slopes, factor of safety, soil mechanics, theory of plasticity, kinematically admissible mechanisms, the Fellenius method

## Abstract

Determining soil and water conditions is essential for designing the optimal foundation and safely transferring loads, including the self-weight of structures, to the ground. Excessive or uneven settlement of the subsoil may ultimately lead to the formation of structural cracks in buildings or the loss of slope stability. In extreme cases, the damage results in structural failure. This paper presents the application of simple solutions from plasticity theory—an evaluation of the upper and lower bounds of the exact solution—to estimate the slope safety factor. It is demonstrated that simple kinematically admissible mechanisms for the non-associated flow rule provide solutions are close to those obtained from the traditional Fellenius method.

## 1. Introduction

Slope stability is one of the issues related to geotechnical design. It is particularly relevant when the building is founded on a site with a significant slope or one is in its immediate vicinity. In such cases, there is a danger that the slope may slip together with the building or trigger a landslide onto the building, which may lead to its damage or even structural failure. Slope failures can also be caused by the disruption of the slope’s natural stability (e.g., earthquake-induced landslides), which can result in property damage, injury, loss of human life, as well as contamination and other serious environmental problems [1,2,3]. In the context of preventing these accidents, it is of great importance to assess the safety factor.

The current standard, Eurocode 7 (EN 1997-1) [4], does not include a single selected computational approach according to which the stability of slopes should be checked. However, it is recommended to check for the total moment and vertical stability of the wedge of detachment. Instead, it is stated what to pay attention to when adopting a particular calculation method and what equilibrium equations it verifies. In addition, in the case of not verifying the equilibrium/horizontal stability, it is recommended to assess the horizontal direction of forces among different slices of soil. The characteristic features in terms of verified equilibrium states of the models summarized in a paper [5] or analyzed in [6,7,8,9] are helpful in this case. The authors of [5] recommend using Spencer’s method and Bishop’s simplified method for slope analysis. Analyses using these two methods to determine slope stability can be found in many studies, including [10,11,12,13].

Numerous other computational methods can be found in the literature and engineering practice. In addition to the traditional approaches discussed in [5], such as the Fellenius method, Janbu, or Sarma, more advanced computational approaches can be considered, for example, those based on probabilistic methods [14,15,16,17,18,19]; semi-probabilistic methods [20], including the stochastic finite difference method [21]; and those based on the finite element method [10,11,22].

At the same time, it should be noted that, currently, with the development of digital methods, there are more and more computer programs and digital computational models that allow the analysis of even very complex cases, as evidenced by, e.g., [1,2,3].

Despite this development, relatively simple methods are still used and recommended for less complex issues [23].

In the present paper, we discuss applying simple solutions from plasticity theory—with an evaluation of the upper and lower bounds of the exact solution—to estimate the slope safety factor. We demonstrate that simple kinematically admissible solutions for the non-associated flow rule give solutions close to those obtained using the Fellenius method [4].

Critical loads and failure mechanisms in the case of passive earth pressure (earth-moving machinery tools) were analyzed using plasticity theory. Due to the adoption of a rigid-perfectly-plastic model, it is difficult or even impossible to obtain exact solutions that satisfy all the conditions of statics and kinematics. Hence, to estimate admissible loads, statically or kinematically admissible solutions are built as the lower or upper bounds of the exact solution. Since the magnitudes for the exact solution are between the lower and upper bounds, kinematically admissible solutions give the lowest force estimate (upper bound), and statically admissible solutions give the highest force estimate (lower bound). 

Plasticity theory was applied to solve boundary issues for the plane deformation state [24], assuming associated or non-associated flow rules. Experimental results for the pressure problem of walls with different shapes (smooth wall, loader bucket models) in a loose medium (sand) show good agreement with calculations for the non-associated flow rule [25].

Ongoing studies have shown that for a cohesive medium, the kinematically admissible mechanisms [26] describe the kinematics of the failure process quite well for both active and passive pressures. Even complex processes related to the working problem of loader bucket models can be described in the same manner [25,27,28,29]. Associated and non-associated flow rules have been applied to describe such processes. For example, the non-associated flow rule has been used to describe a quite advanced wall pushing process [27,29].

In the present study, plasticity theory and simple kinematically admissible mechanisms were used to evaluate slope stability. The obtained results, easy to calculate, were compared with those obtained using the Fellenius method. The kinematically admissible mechanisms were applied within the landslide area, as determined by the method mentioned above.

## 2. Materials and Methods

The issue of slope stability can be analyzed using plasticity theory and a stress function $G(\sigma_{ij})$ called the plastic potential, where the relationship between strain and stress rates is defined by

(1) $${\dot{\varepsilon }}_{ij}=\lambda \frac{\partial G}{\partial \sigma_{ij}}$$

where λ is a certain scalar multiplier that has a constant value at a given state and point; it has different values at different points.

If the plastic potential is identified with the boundary condition (e.g., Mohr–Coulomb), Equation (1) is called an associated flow rule. If it is a different condition (Tresca condition was adopted in this paper), we deal with a non-associated flow rule.

In the general spatial issue of quasi-static motion, we have 16 unknowns to be determined, and we have 16 equations.

This system is quite challenging to solve, but effective solutions can be obtained for special states, such as the planar flow state or axisymmetric flows.

In the case of planar surface flow, we have:

▪ Two equilibrium equations

(2) $$\frac{\partial \sigma_{x}}{\partial x}+\frac{\partial \tau_{xy}}{\partial y}=0$$

(3) $$\frac{\partial \sigma_{y}}{\partial y}+\frac{\partial \tau_{xy}}{\partial x}=\gamma$$

where γ is a volumetric weight;

▪ a limit state condition, e.g., Mohr–Coulomb;
▪ The relationships between the components of the stress vector and tensor, which take the form:

(4) $$\frac{{\dot{\varepsilon }}_{x}}{\frac{\partial G}{\partial \sigma_{x}}}=\frac{{\dot{\varepsilon }}_{y}}{\frac{\partial G}{\partial \sigma_{y}}}=\frac{{\dot{\varepsilon }}_{xy}}{\frac{\partial G}{\partial \tau_{xy}}}$$

**Figure 1:**
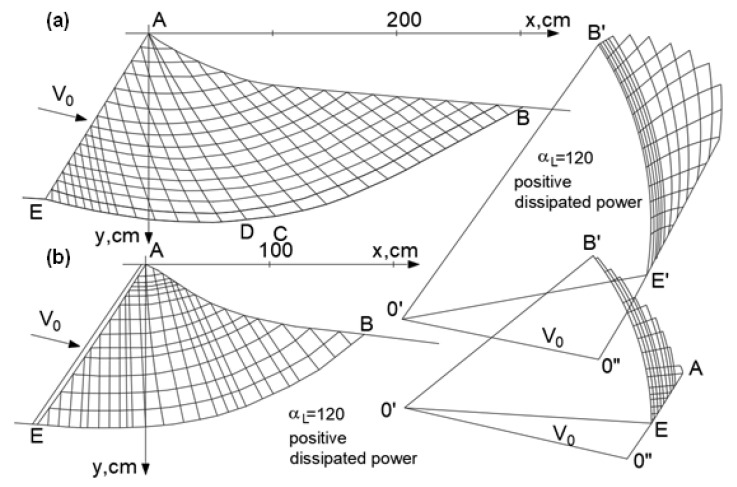
Velocity grids and the corresponding hodograph for the problem of wall pressure in a Coulomb medium for (**a**) associated flow rule and (**b**) non-associated flow rule.

A practical method of solving plane flow problems involves first determining the state of stress and then the velocity field. Figure 1 shows an example of its application to the analysis of smooth wall pressure (passive pressure).

Despite the simplifications, these solutions are labor-intensive and have limited practical application. In addition, adopting a rigid-perfectly-plastic model makes it difficult to obtain exact solutions, especially specific solutions that satisfy all the conditions of statics and kinematics. Hence, statically or kinematically admissible solutions are built as approximations, which, for the associated flow rule, represent the lower bound (statically admissible solutions) and upper bound (kinematically admissible solutions) to the exact solution. The solution is located between these quantities.

The upper bound means that, for example, the allowable loads are no greater than those specified, and the lower bound is not less than that specified. The smaller the difference between these bounds, the closer to the exact solution we are.

In the case of the non-associated flow rule, there is no evidence for the limit analysis theorems, but we proceed via analogy.

**Figure 2:**
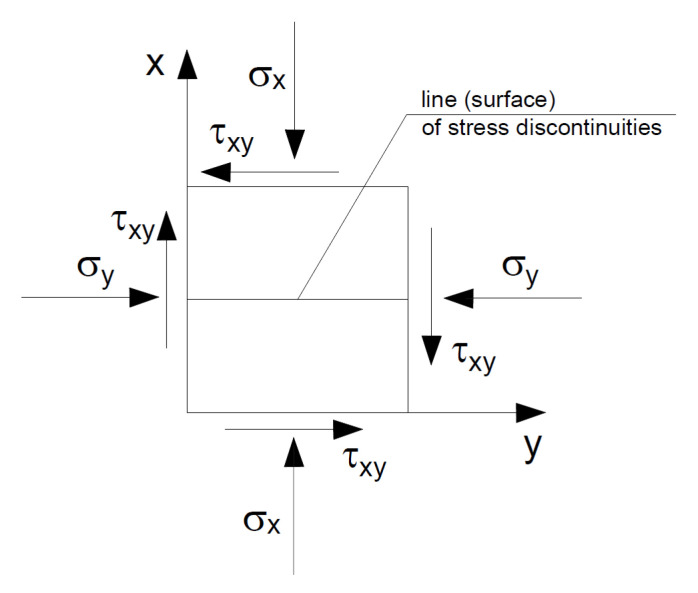
Surface of stress discontinuity.

In the statically admissible solution, static conditions must be satisfied, but this requirement does not apply to kinematic conditions [30], which allows determining lower stress values and searching for the solution giving the highest values [30]. The solution is based on the analysis of separated slices of, for example, a slope and the determination of their interaction by checking the state of stress. In each slice, the values of forces (loads) are determined, as a result of which plasticization of the material can occur based on, for example, the Mohr–Coulomb condition. The conditions that the surfaces (lines) of discontinuous stresses must meet are derived from the equilibrium conditions (Figure 2).

From this, it follows that the stresses on both sides of the discontinuity surface must be the same in such a structure. The stress parallel to this line may experience a spike.

On the other hand, the upper limit of load values can be determined on the basis of kinematically admissible mechanisms arbitrarily adopted for the system. To apply a kinematically admissible solution, the mechanism adopted for consideration must meet the following criteria:

- The continuity of the medium must be achieved.
- The rule of flow must be assumed.
- Boundary conditions [30] must be met.

Static conditions need not be satisfied in this case.

In addition, since this is not an exact solution and depends on the kinematic mechanism adopted by the researcher, the solution with the smallest value of estimated forces (loads) that is “closer” to the exact solution should be searched for. The kinematically admissible solution (the number of mechanisms is practically infinite) assumes that in the medium under consideration, deformation occurs along the lines of discontinuity separating areas of different kinematics defined by the researcher. 

Consider an element undergoing plane deformation, which consists of the movement with two rigid blocks moving along the discontinuity line L.

**Figure 3:**
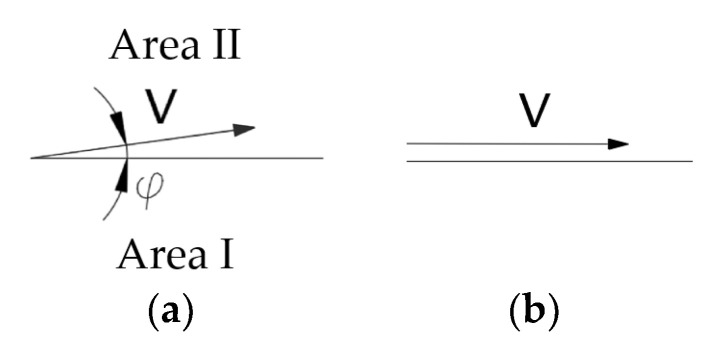
Line of discontinuity of velocity: (**a**) associated flow rule and (**b**) non-associated flow rule.

In the case of a Mohr–Coulomb medium and the associated flow rule, the velocity increase vector along the discontinuity line deviates from it by an angle φ (internal friction) (Figure 3a).

In the case of a Mohr–Coulomb medium and the non-associated flow rule (we assume the Tresca potential), the vector of the velocity increase on the discontinuity line is tangent to it (Figure 3).

It can be demonstrated that the unit energy dispersion $\dot{D}$ on the discontinuity line is determined by the correlation:

(5) $$\dot{D}=c\cdot V\cdot cos\varphi$$

where *c* is cohesion for the associated flow rule, and:

(6) $$\dot{D}=c\cdot V$$

for the non-associated flow rule.

In the general case, it is possible to distinguish some typical mechanisms relating to the adopted division of the embankment or slope, along with the adopted behavior of the separate parts and the planes of their mutual displacement, as described in more detail in [24,30].

Regardless of the mechanism adopted, a hodograph—a velocity plan incorporating velocity vectors related to a common pole—should be made in each case, using the properties of the velocity field [30]. 

**Figure 4:**
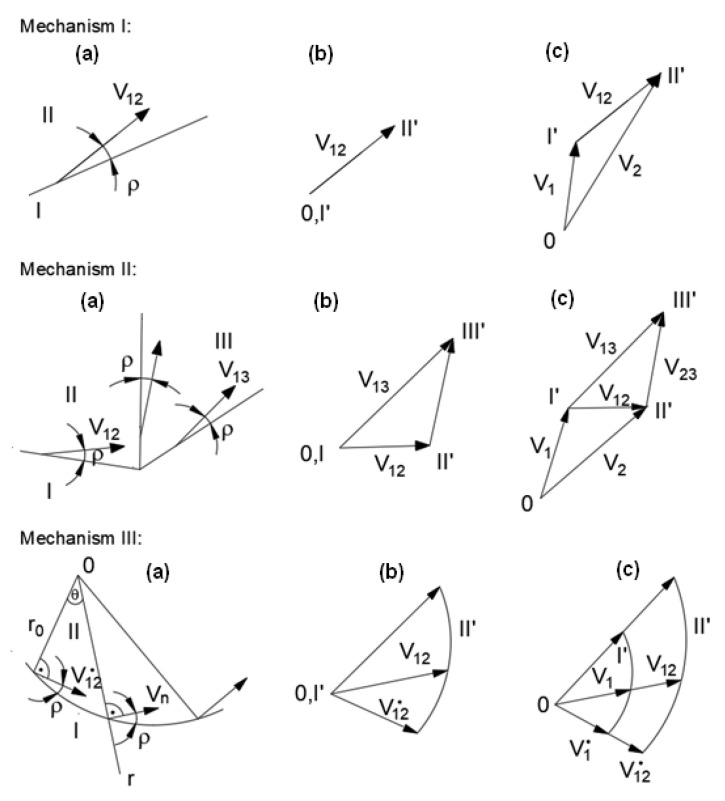
Examples of kinematic mechanisms: (**a**) slip mechanism and (**b**,**c**) speed hodographs.

Figure 4 shows three simple kinematically admissible mechanisms along with velocity hodographs for the associated flow rule.

The solution for determining the force required to implement the adopted kinematic mechanism can be found by applying the block equilibrium method or the energy method. 

The first method involves determining the forces acting on the various parts of the considered system, and the graphical or mathematical solution is based on the estimation of the unknown force values. The energy method involves determining the maximum force based on the energy balance of the system under consideration.

An example of the application of kinematically admissible methods can be found in the work of [1], which analyzed the passive pressure acting on the retaining wall, which results from the operation of machinery during earthworks, as well as in the work of [31], in which the stability of slopes was estimated based on the analysis of the equilibrium of curved slices.

## 3. Results

### 3.1. Statically and Kinematically Admissible Solution—Simple Example

As mentioned in Section 2, to calculate the upper and lower bounds of the exact solution, both statically and kinematically admissible solutions can be constructed. The concept of such simple solutions for a vertical slope is presented below. 

#### 3.1.1. Statically Admissible Solution—Lower Bound

**Figure 5:**
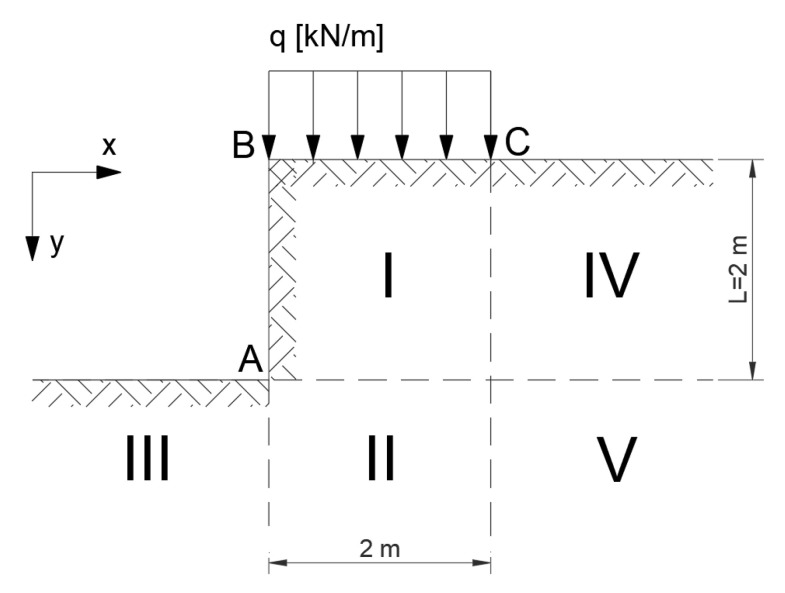
Vertical slope divided into areas.

It is assumed that in the areas shown in Figure 5, the state of stress is determined by Equations (7)–(11). These relationships must be statically admissible and meet the conditions of discontinuity of stress—dashed lines indicate the lines of discontinuity of stress (Figure 2). 

There is no load along line AB and $in\;Areas\;I\;and\;IV,\;it\;was\;assumed\;that\;\sigma_{x}=0.$ In Areas III, II, and V, horizontal stress $\sigma_{x}$ has to be equal and it was assumed that $\sigma_{x}=\gamma \left(y-L\right).$ In all areas, stresses are statically admissible, and conditions of discontinuity of stress between areas are fulfilled.

Area I:

(7) $$\begin{array}{cc} \sigma_{y}=q+\gamma y & \sigma_{x}=0 \end{array}$$

Area IV:

(8) $$\begin{array}{cc} \sigma_{y}=\gamma y & \sigma_{x}=0 \end{array}$$

Area III:

(9) $$\begin{array}{cc} \sigma_{y}=\gamma (y-L) & \sigma_{x}=\gamma (y-L) \end{array}$$

Area II:

(10) $$\begin{array}{cc} \sigma_{y}=q+\gamma y & \sigma_{x}=\gamma (y-L) \end{array}$$

Area V:

(11) $$\begin{array}{cc} \sigma_{y}=y\gamma & \sigma_{x}=\gamma (y-L \end{array})$$

It can be noticed that Area I gives the most unsafe results. 

**Figure 6:**
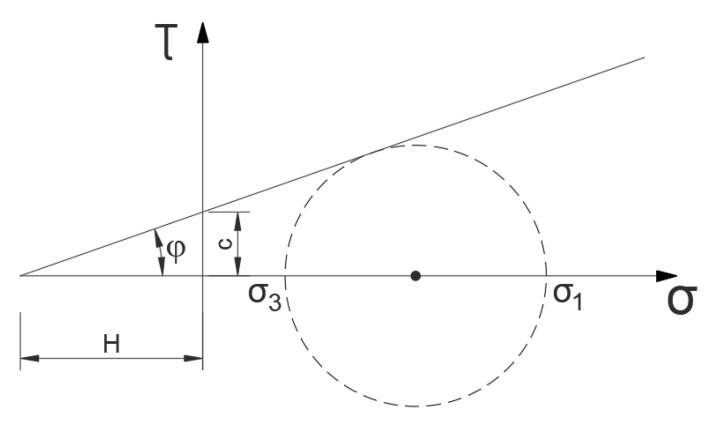
Mohr–Coulomb condition.

The results of the use of the Mohr–Coulomb failure criterion are shown in Figure 6.

(12) $$\left(\sigma_{1}-\sigma_{3}\right)=\left(\sigma_{1}+\sigma_{3}+2H\right)\sin\varphi \;\equiv \left(\sigma_{1}-\sigma_{3}\right)=\left(\sigma_{1}+\sigma_{3}+2\frac{c}{\tan\varphi }\right)sin\varphi$$

where *c* is cohesion, and *φ* is the internal friction angle.

(13) $$q=\frac{2Hsin\varphi +(sin\varphi -1)\gamma y}{1-sin\varphi }$$

For a slope made of a single soil type, whose angle of internal friction is φ = 20°, with cohesion *c* = 20 kPa, volume density $\rho =2.15\frac{g}{{cm}^{3}}$, and volume weight $\gamma =21.5\frac{kN}{m^{3}}$,

$$q=14.13\frac{kN}{m},\;\;\;P=2\cdot q=28.26\;kN$$

The safety factor for unloaded slope *q* = 0.

(14) $$\sigma_{1}=\gamma L$$

(15) $$\sigma_{2}=0$$

(16) $$F=\frac{\sigma_{dop}}{\sigma }=1.33$$

where ${\sigma \;}_{dop}$ is the allowable stress, and *φ* is the internal friction angle.

It can be demonstrated that the stress state in Areas III, II, and V for *q* < *q_dop_*, where *q_dop_* is an allowable load, does not reach an arbitrarily deep boundary condition (at infinity).

#### 3.1.2. Kinematically Admissible Solution—Associated Flow Rule—Upper Bound

Energy dissipation for the associated flow rule, where P=q·BC,

$$G=0.5\cdot \left|BC\right|\cdot \left|BA\right|\cdot \gamma$$

$$V_{0}\left(y+P\right)=V_{1}\cdot sin25{}^{\circ}\cdot AC\cdot c$$

(17) $$V_{0}=V_{1}\cdot sin25{}^{\circ}$$

and the energy equilibrium condition is defined by the relation:

(18) $$V_{0}\left(G\right)=V_{1}\cdot cos\varphi \cdot c\cdot AC$$

Hence, for the data adopted:

$$q=41.43\frac{kN}{m}$$

The safety factor (for the unloaded slope) is defined as the ratio of the energy dispersed at the slip line to the energy of the sliding block.

(19) $$F=\frac{V_{1}\cdot cos\varphi \cdot AC\cdot c}{V_{0}(G)}=2.93$$

**Figure 7:**
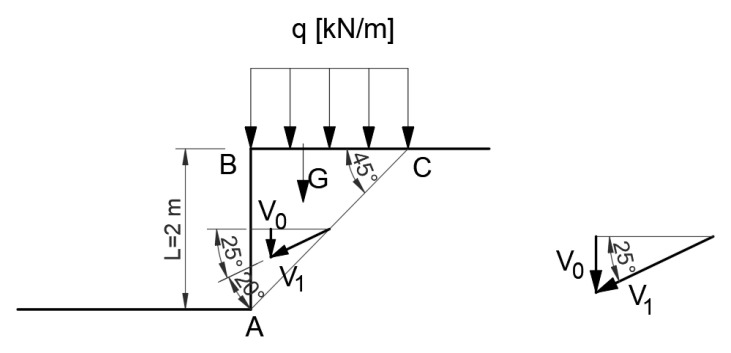
Diagram of force distribution according to the associated flow rule.

To calculate the forces and the safety factor, the block balance method (Figure 7), equivalent to the energy method, or a simpler method, i.e., energy dissipation, can be used.

#### 3.1.3. Kinematically Admissible Solution—Non-Associated Flow Rule—Lower Bound 

From the energy equilibrium condition,

(20) $$V_{0}\left(G+P\right)=V_{1}\cdot AC$$

Hence, for the data adopted:

$$q=18.52\frac{kN}{m}$$

The safety factor for unloaded slope is:

(21) $$F=\frac{V_{1}\cdot c\cdot AC}{V_{0}(G)}=1.86$$

Table 1 summarizes the load values and safety factors estimated using the three calculation methods.

**Figure 8:**
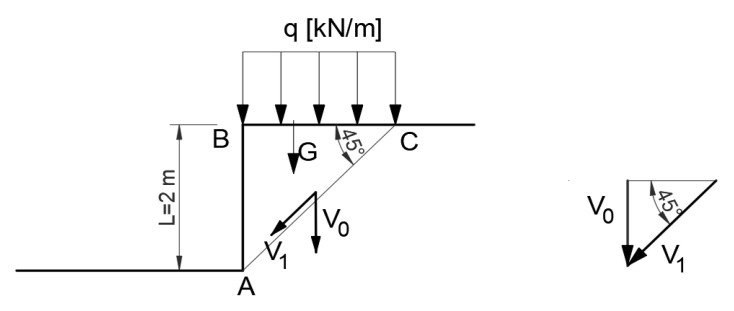
Diagram of the distribution of forces according to the non-associated flow rule.

The distinction between the non-associated (Figure 8) and associated flow rule (Figure 7) exists in the intrinsic property of a material and has to be established/assumed. Not knowing this material property, to describe the slope sliding process, both rules were applied, and safety factors were calculated for comparison. In the case of material described using a non-associated flow rule, a solution closer to a statically admissible solution is obtained, which is thus closer to a precise solution.

### 3.2. Simple Kinematically Admissible Solutions for the Slope Stability Problem—Comparison with the Classical Fellenius Method

#### 3.2.1. Slope Stability—The Fellenius Method

As the first computational example, the bearing capacity of a slope was analyzed, inclined at an angle of 60° and built of a single type of soil (clayed sand), with angle of internal friction *φ* = 20°, cohesion *c* = 20 kPa, and volumetric density $\rho =2.15\frac{g}{{cm}^{3}}$.

**Figure 9:**
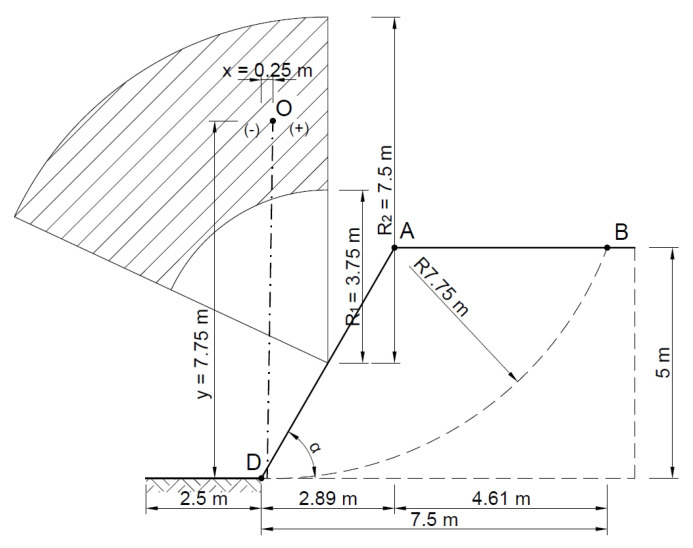
Diagram of the analyzed slope.

Figure 9 shows a diagram of the analyzed slope, where, according to the Fellenius method, the point of theoretical rotation O is marked, with the following coordinates *x* = 0.25 m and *y* = 7.75 m, and the cylindrical slip plane is plotted.

**Figure 10:**
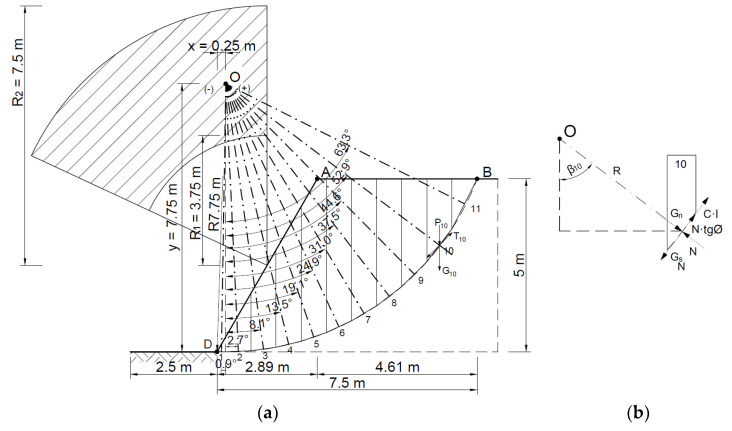
(**a**) Division of the slope into slices with a diagram of the forces acting on a slice; (**b**) distribution of forces in a single slice.

The area thus defined was divided into 11 (Figure 10) slices with a width B of no more than 0.73 m according to Equation (1):

(22) B≤0.1·R

where *B* is the width of the block (slice); *R* is the radius of rotation of the block.

The values of the forces acting on each block (slice) are determined using Equations (2)–(5).

(23) $$G_{i}=P_{i}\cdot \gamma$$

where *G_i_* is the weight of a single block, *P_i_* is the area of a separate block, and *γ* is the volumetric weight of the soil (21.9 kN/m^3^).

(24) $$N_{i}=G_{ni}=G_{i}\cdot \cos\alpha_{i}$$

*N_i_* is the normal component. cos *α_i_* is the cosine of angle alpha.

(25) $$S_{ti}=c\cdot l_{i}+N_{i}\cdot \mathrm{tg}\phi$$

*c* is cohesion; *l_i_* is the length of the section of contact between the block and the base; tg⁡ϕ is the tangent of the internal friction angle.

(26) $$G_{si}=G_{i}\cdot \sin\alpha_{i}$$

The holding moment *M_u_* and the moment of causing rotation *M_o_* are given by the following correlation:

(27) $$M_{u}=\sum_{i=1}^{n}\left(r\cdot S_{ti}\right)=\sum_{i=1}^{n}\left(r\cdot \left(N_{i}\cdot \mathrm{tg}\phi +c\cdot l_{i}\right)\right)=2555.6\;kNm$$

(28) $$M_{o}=\sum_{i=1}^{n}\left(r\cdot G_{i}\cdot \sin\alpha_{i}\right)=1514.6\;kNm$$

Hence, the slope safety factor *F* (7) is:

(29) $$F=\frac{M_{u}}{M_{o}}=1.7>1.0$$

Thus, the slope can be considered stable.

#### 3.2.2. Kinematically Admissible Solution—One Slip Line Associated Flow Rule 

An analysis was also conducted for a simpler case, where the slope landslide was modeled as a single block (for which the parameters are summarized in Table 2). In this case, too, both associated and non-associated flow rules were used.

**Figure 11:**
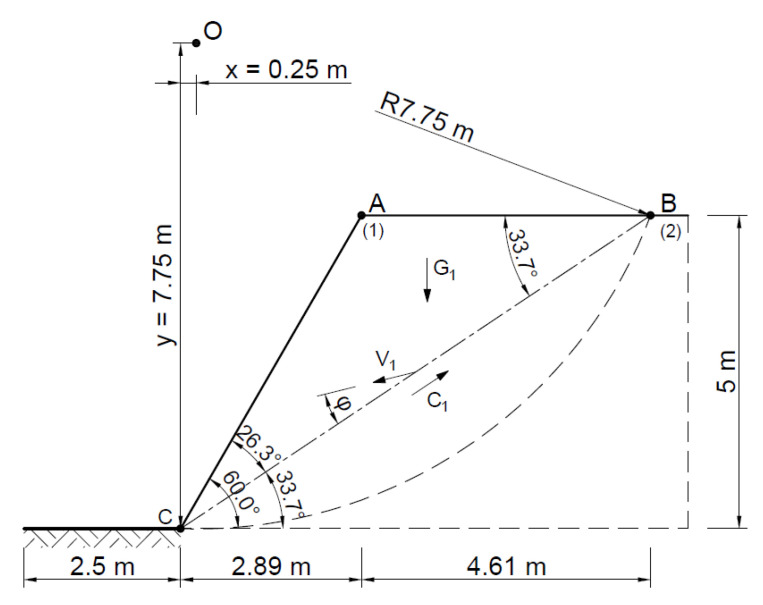
Distribution of forces and velocities acting on the slip line (associated flow rule).

**Figure 12:**
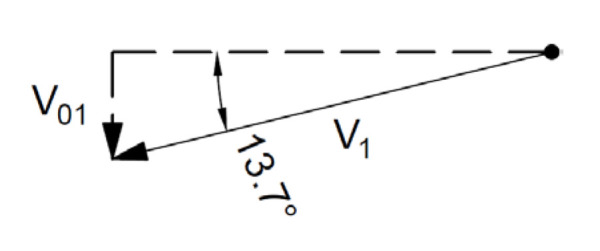
Velocity hodograph for associated flow rule.

For the associated flow rule, the distribution of forces and velocities and hodograph are shown in Figure 11 and Figure 12.

(30) $$V_{01}=V_{1}\cdot \sin\left(\alpha \right)$$

Hence:

(31) $$F_{o}=G_{1}\cdot V_{01}=59.5\;kN\cdot V_{1}$$

(32) $$F_{u}=\left|BC\right|\cdot c\cdot V_{1}\cdot \cos\phi =169.2\;kN\cdot V_{1}$$

(33) $$F=\frac{F_{u}}{F_{o}}=\frac{169.2\;kN\cdot V_{1}}{59.5\;kN\cdot V_{1}}=2.8$$

(34) $$F_{u}\geq F_{o}$$

#### 3.2.3. Kinematically Admissible Solution—One Slip Line (Non-Associated Flow Rule)

**Figure 13:**
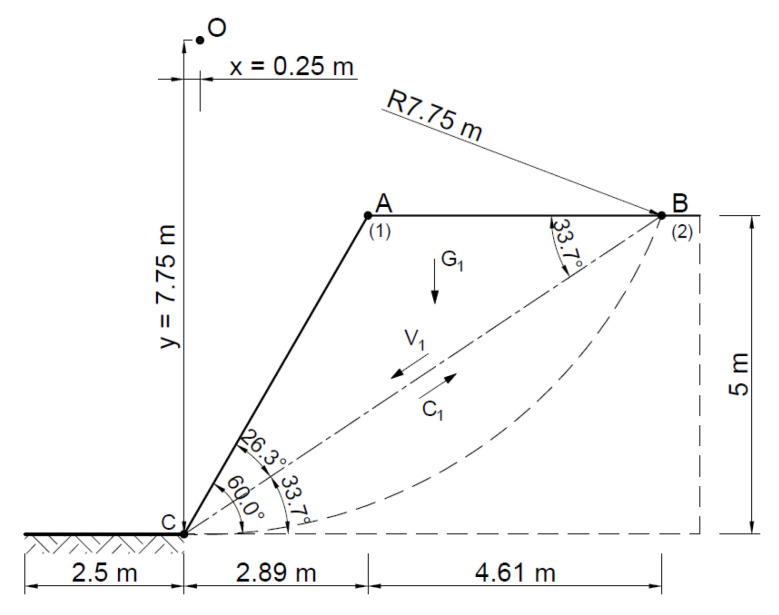
Distribution of velocities acting in the slope according to the adopted kinematically admissible solution (non-associated flow rule).

**Figure 14:**
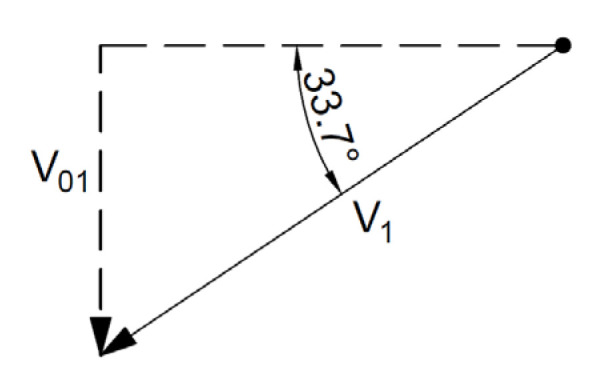
Velocity hodograph for non-associated flow rule.

For the non-associated flow rule, the velocity distribution and the hodograph are shown in Figure 13 and Figure 14.

(35) $$V_{01}=V_{1}\cdot \sin\left(\alpha \right)=0.55\cdot V_{1}$$

Hence:

(36) $$F_{o}=G_{1}\cdot V_{01}=136.4\;kN\cdot V_{1}$$

(37) $$F_{u}=\left|BC\right|\cdot c\cdot V_{1}=180\;kN\cdot V_{1}$$

(38) $$F=\frac{F_{u}}{F_{o}}=\frac{180\;kN\cdot V_{1}}{136.4\;kN\cdot V_{1}}=1.3$$

#### 3.2.4. Kinematically Admissible Solution for an Associated Flow Rule

The stability analysis of the selected slope was also carried out by assuming two rigid blocks as the failure mechanism. The area at risk of the landslide was assumed to be as in the Fellenius method, entering the rigid area—the triangle ∆*ABC* and ∆*ACD*.

**Figure 15:**
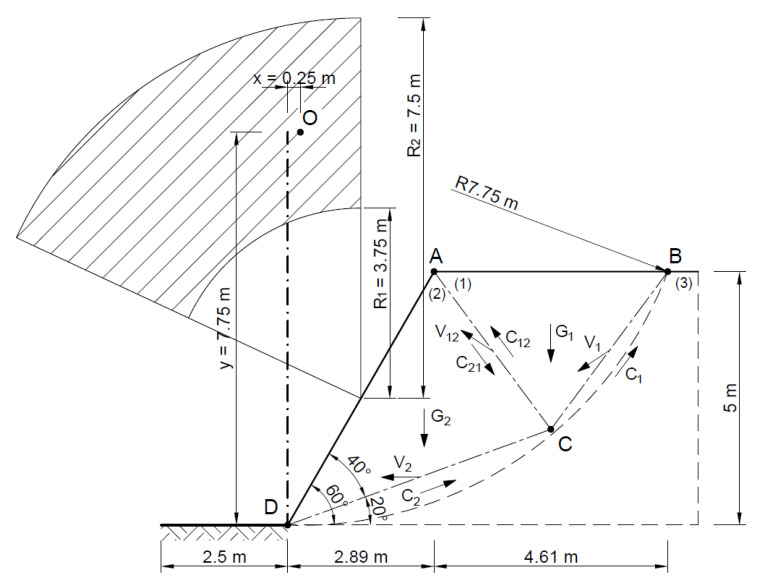
Dividing the slope into blocks for a kinematically admissible solution.

**Figure 16:**
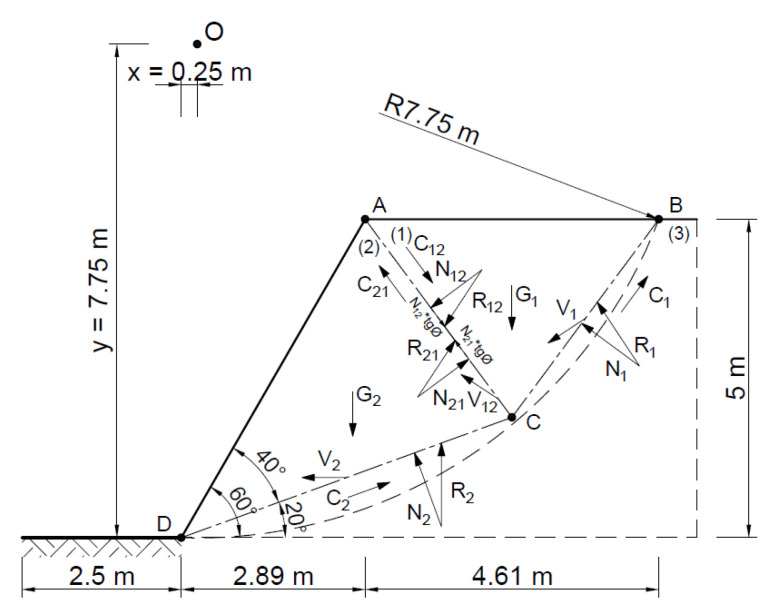
Distribution of forces acting in the slope according to the adopted kinematically admissible solution.

Figure 15 and Figure 16 show the slip lines and velocities of each block and the forces acting on the slip lines.

**Figure 17:**
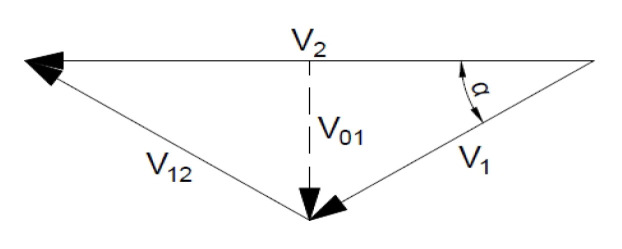
Velocity hodograph for associated flow rule with two rigid blocks.

Figure 17 shows the hodograph where for the associated flow rule *α* = 33.4°.

The geometric parameters of the adopted fields are given in Table 3.

The energy method was used to determine the safety of the slope.

(39) $$F_{u}=\sum_{i=1}^{n}\left(L_{i}\cdot c\cdot V_{i}\cdot \cos\varphi \right)$$

(40) $$F_{o}=\sum_{i=1}^{n}\left(G_{i}\cdot V_{0i}\right)$$

(41) $$V_{1}=V_{12}$$

(42) $$V_{2}=2\cdot V_{1}\cdot \cos\left(\alpha \right)=1.67{\cdot V}_{1}$$

(43) $$V_{01}=V_{1}\cdot \sin\left(\alpha \right)={0.55\cdot V}_{1}$$

(44) $$V_{02}=0$$

Hence,

(45) $$F_{o}=G_{1}\cdot V_{01}+G_{2}\cdot V_{02}=84.8\;kN\cdot V_{1}$$

(46) $$F_{u}=\left|BC\right|\cdot c\cdot V_{1}\cdot \cos\phi +\left|AC\right|\cdot c\cdot V_{12}\cdot \cos\phi +\left|CD\right|\cdot c\cdot V_{2}\;\cdot \cos\phi =286.17\;kN\cdot V_{1}$$

(47) $$F_{u}\geq F_{o}$$

(48) $$F=\frac{F_{u}}{F_{o}}=3.4$$

#### 3.2.5. Kinematically Admissible Solution for a Non-Associated Flow Rule 

**Figure 18:**
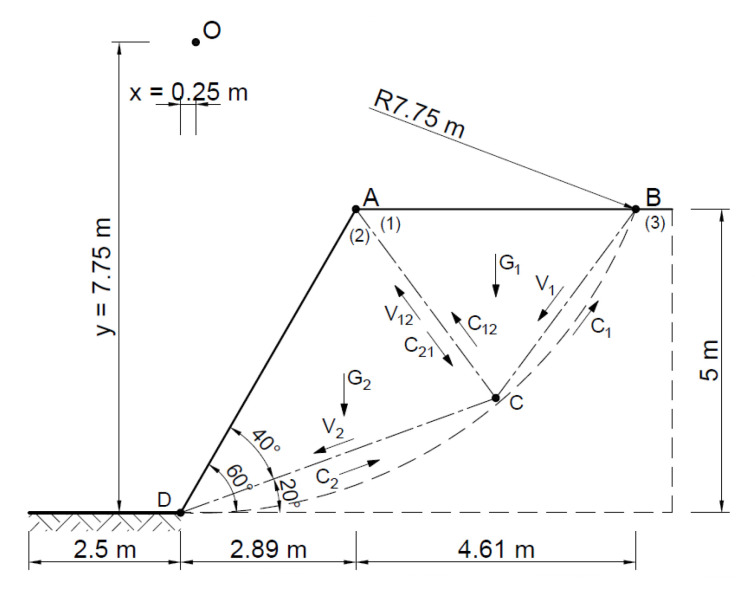
Distribution of velocities acting in the slope.

**Figure 19:**
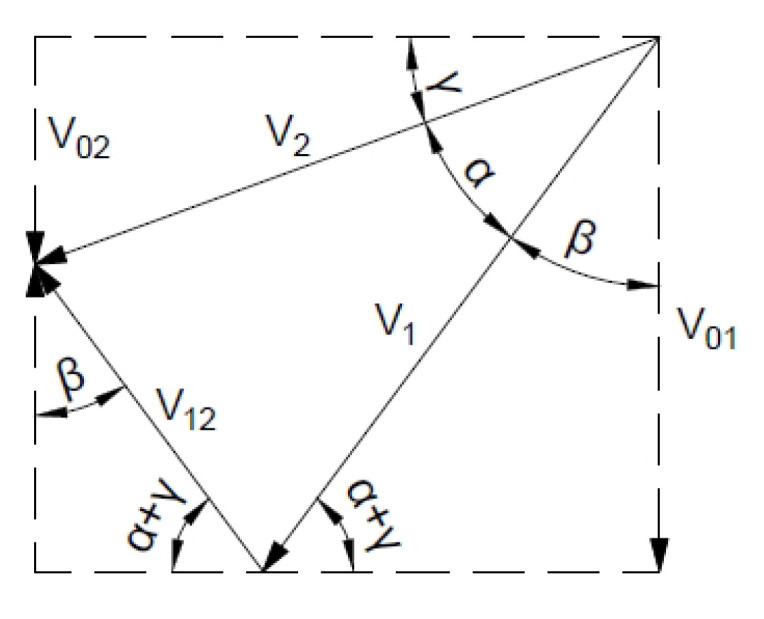
Velocity hodograph for non associated flow rule with two rigid blocks.

Figure 18 shows the velocity distribution on the *BC*, *AC*, and *DC* slip lines, and Figure 19 shows the hodograph.

(49) $$F_{u}=\sum_{i=1}^{n}\left(L_{i}\cdot c\cdot V_{i}\cdot \cos\phi \right)$$

(50) $$F_{o}=\sum_{i=1}^{n}\left(G_{i}\cdot V_{0i}\right)$$

(51) $$V_{01}=V_{1}\cdot cos\left(\gamma \right)=0.80\cdot V_{1}$$

(52) $$V_{2}=V_{1}\cdot \frac{sin\left(\gamma \right)}{cos\left(\alpha \right)}\cdot \left(1+\frac{cos\left(\gamma +\alpha \right)}{cos\left(\gamma -\alpha \right)}\right)=0.999\cdot V_{1}$$

(53) $$V_{02}=V_{2}\cdot sin\left(\alpha \right)=0.34\cdot V_{1}$$

(54) $$V_{12}=V_{1}\cdot \frac{cos\left(\gamma +\alpha \right)}{cos\left(\gamma -\alpha \right)}=0.57\cdot V_{1}$$

Hence:

(55) $$F_{o}=G_{1}\cdot V_{01}+G_{2}\cdot V_{02}=198.4\;kN\cdot V_{1}$$

(56) $$F_{u}=\left|BC\right|\cdot c\cdot V_{1}+\left|AC\right|\cdot c\cdot V_{12}+\left|CD\right|\cdot c\cdot V_{2}=234.0\;kN\cdot V_{1}$$

(57) $$F=\frac{F_{u}}{F_{o}}=\frac{234.0\;kN\cdot V_{1}}{198.4\;kN\cdot V_{1}}=1.2$$

#### 3.2.6. Kinematically Admissible Solution—Associated Flow Rule

The stability analysis of the selected slope was also carried out using a system of four blocks in the rigid area—the triangles ∆*ABC*, ∆*ACD*, ∆*ADE*, and ∆*AEF*.

**Figure 20:**
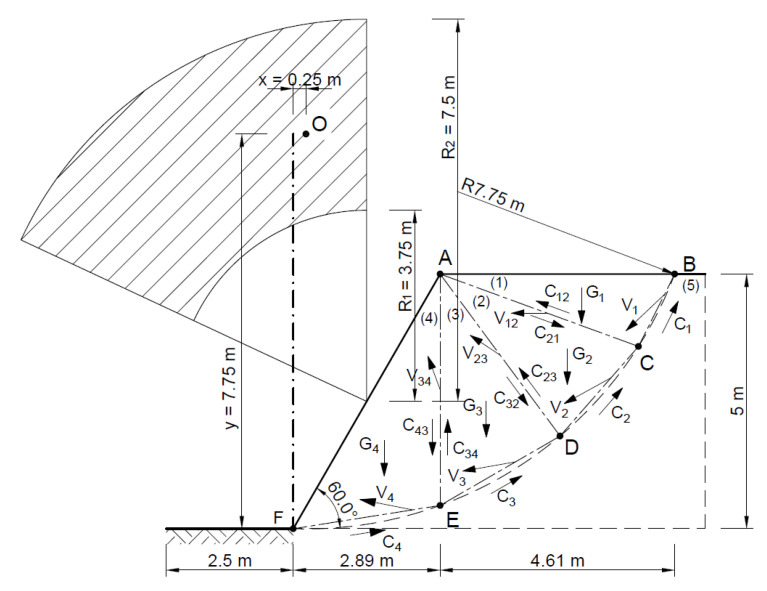
Distribution of forces acting in the slope according to the adopted kinematically admissible solution.

Figure 20 shows the slip lines and velocities of each block, as well as the forces acting on the slip lines, whose geometric parameters for the associated flow rule are given in Table 4.

**Figure 21:**
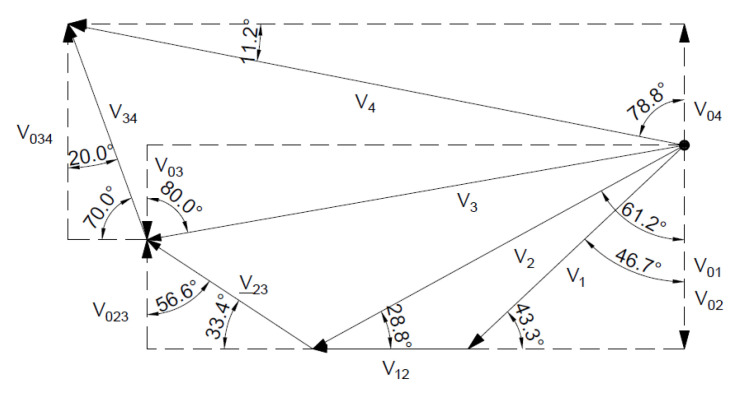
Velocity hodograph for associated flow rule with tree rigid blocks.

Figure 21 shows the velocity hodograph. 

The energy method was used to determine the safety of the slope.

(58) $$F_{u}=\sum_{i=1}^{n}\left(L_{i}\cdot c\cdot V_{i}\cdot \cos\varphi \right)$$

(59) $$F_{o}=\sum_{i=1}^{n}\left(G_{i}\cdot V_{0i}\right)$$

(60) $$F_{u}\geq F_{o}$$

(61) $$F=\frac{F_{u}}{F_{o}}=2.2$$

#### 3.2.7. The Kinematically Admissible Solution—The Associate Flow Rule 

**Figure 22:**
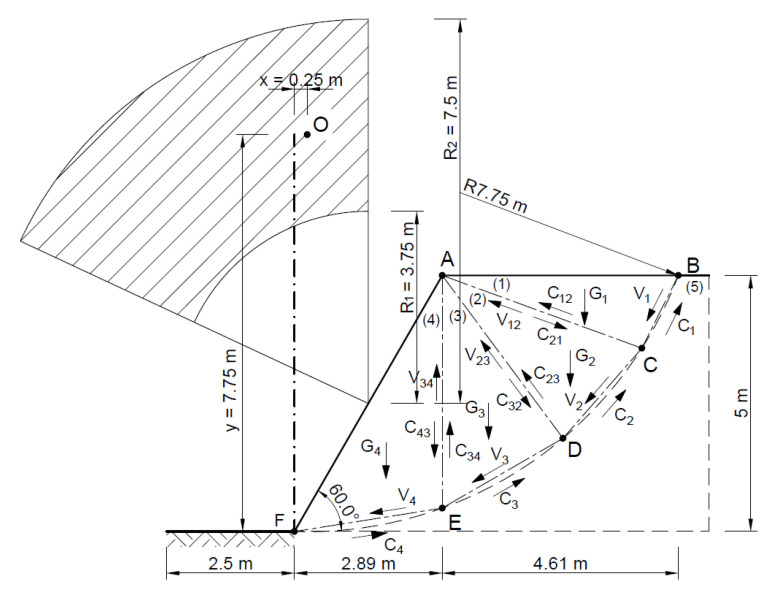
Distribution of forces acting in the slope according to the adopted kinematically admissible solution.

An analysis was also performed for the non-associated flow rule. Figure 22 shows the slip and velocity lines of each block and the forces acting on the slip lines.

**Figure 23:**
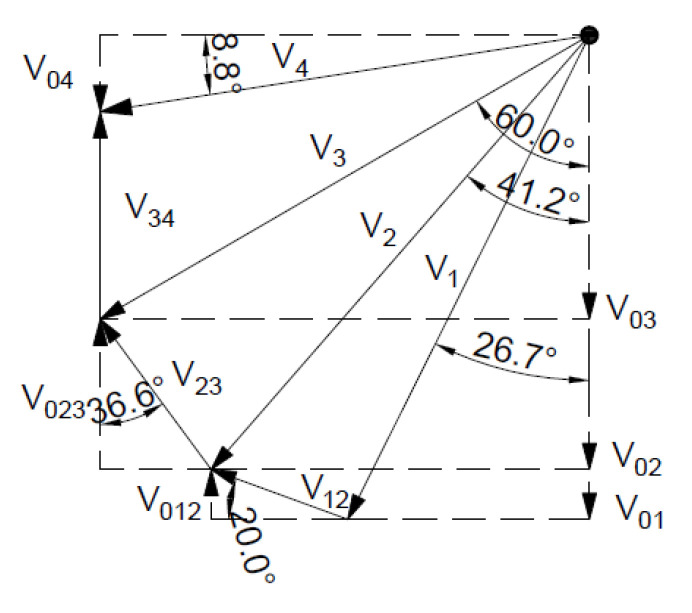
Velocity hodograph for non associated flow rule with tree rigid blocks.

Figure 23 shows the hodograph of velocity for the non-associated flow rule. 

The energy method was used to determine the safety of the slope.

(62) $$F_{u}=\sum_{i=1}^{n}\left(L_{i}\cdot c\cdot V_{i}\right)$$

(63) $$F_{o}=\sum_{i=1}^{n}\left(G_{i}\cdot V_{0i}\right)$$

(64) $$F_{u}\geq F_{o}$$

(65) $$F=\frac{F_{u}}{F_{o}}=1.3$$

A summary of the results is presented in the Table 5.

As mentioned before, the distinction between the non-associated and associated flow rule exists in the intrinsic property of a material and has to be established/assumed. Not knowing such material property, to describe the slope sliding process, both rules were applied, and safety factors were calculated for comparison.

In this paper, where the Mohr–Coulomb medium is discussed, we assume the Tresca potential for the non-associated flow rule. It is worth mentioning that potentials other than the Tresca potential can be taken into account to describe material behavior. 

In the case of the Fellenius method, the safety factor was built in terms of the moment ratio, since the “block rotation” mechanism was assumed, and the comparison of the holding moment and moment-rotation is most appropriate.

In the case of kinematically admissible mechanisms, the mechanism of sliding blocks is assumed, and the comparison of work rate was used to calculate the safety factor. 

## 4. Conclusions

This paper presents the application of simple plasticity theory solutions that give a basic assessment of slope stability and bearing capacity for simple cases. Using very simple kinematically admissible solutions, it is easy to assess the stability or bearing capacity of a slope. The calculations presented in Section 3.1 show that for a material described using the non-associated flow rule, the solution is close to that obtained with the Fellenius method. This way one can have a simple and quick rough estimation of slope stability.

Even a very simple kinematically admissible mechanism (one sliding block), inscribed in the landslide area determined with the Fellenius method, can be taken into consideration (Table 5).

The authors plan to carry on further research on this subject, including experimental studies.

## Figures and Tables

**Table 1:** Load values and safety factors obtained using three calculation methods.

Method	A Statically Admissible Solution—Lower Bound	Associated Flow Rule—Upper Bound	Non-Associated Flow Rule—Upper Bound
Safety factor	1.33	2.93	1.86

**Table 2:** Parameters of the separated block.

Block	Area *P_i_* (m^2^)	Side lengths *L_i_* (m)			$\mathrm{Weight}\;G=P_{i}\cdot$*γ* (kN)	Tangential Component C=c·*L_i_* (kN)
*ABC*	11.5	|*AB*| = 4.6	|*AC*| = 5.8	|*BC*| = 9.0	248	c·|*BC*| = 180 kN

**Table 3:** Geometric parameters of the adopted fields.

Block	Area *P_i_* (m^2^)	Side Lengths *L_i_* (m)			$\mathrm{Weight}\;G=P_{i}\cdot$*γ* (kN)	Tangential Component C=c·*L_i_* (kN)
*ABC*	7.17	|*AB*| = 4.61	|*AC*| = 3.87	|*BC*| = 3.87	154.2	c·|AC|=c·|*BC*|= 77.4 kN
*ACD*	10.25	|*AD*| = 5.77	|*AC*| = 3.87	|*CD*| = 5.52	220.4	c·|*CD*|= 110.4 kN

**Table 4:** Geometric parameters of adopted fields.

Block	Field *P_i_* (m^2^)	Side Lengths *L_i_* (m)			$\mathrm{Weight}\;G=P_{i}\cdot$*γ* (kN)	Tangential Component C=c·*L_i_* (kN)
*ABC*	3.27	|*AB*| = 4.61	|*AC*| = 4.15	|*BC*| = 1.59	70.3	*c*·|*AC*| = 83.0 kN *c*·|*BC*| = 31.8 kN
*ACD*	4.53	|*AC*| = 4.15	|*AD*| = 3.96	|*CD*| = 2.34	97.4	*c*·|*AD*| = 79.2 kN *c*·|*CD*| = 46.8 kN
*ADE*	5.36	|*AD*| = 3.96	|*AE*| = 4.54	|*DE*| = 2.72	115.2	*c*·|*AE*| = 90.8 kN *c*·|*DE*| = 54.4 kN
*AEF*	6.56	|*AE*| = 4.54	|*AF*| = 5.77	|*EF*| = 2.92	141.0	*c*·|*EF*| = 58.4 kN *c*·|*AE*| = 90.8 kN

**Table 5:** Summary of the test results.

Used Method		Safety Factor
Fellenius Method		$F=\frac{M_{u}}{M_{o}}=1.7$
Kinematically admissible solution—one block	non-associated flow rule	$F=\frac{F_{u}}{F_{o}}=1.3$
	associated flow rule	$F=\frac{F_{u}}{F_{o}}=2.8$
Kinematically admissible solution—two blocks	non-associated flow rule	$F=\frac{F_{u}}{F_{o}}=1.2$
	associated flow rule	$F=\frac{F_{u}}{F_{o}}=3.4$
Kinematically admissible solution—four blocks	non-associated flow rule	$F=\frac{F_{u}}{F_{o}}=1.3$
	associated flow rule	$F=\frac{F_{u}}{F_{o}}=2.2$

## Data Availability

Not applicable.
